# Rapid identification of Bordetella pertussis pertactin gene variants using LightCycler real-time polymerase chain reaction combined with melting curve analysis and gel electrophoresis.

**DOI:** 10.3201/eid0706.010606

**Published:** 2001

**Authors:** J Mäkinen, M K Viljanen, J Mertsola, H Arvilommi, Q He

**Affiliations:** Finnish Pertussis Reference Laboratory, National Public Health Institute, Department in Turku, Finland. johanna.makinen@utu.fi

## Abstract

Recently, eight allelic variants of the pertactin gene (prn1-8) have been characterized in Bordetella pertussis strains isolated in Europe and the United States. It has been suggested that the divergence of the pertactin types of clinical isolates from those of the B. pertussis vaccine strains is a result of vaccine-driven evolution. Sequencing of the prn, which is relatively time-consuming, has so far been the only method for the differentiation of prn types. We have developed a rapid real-time polymerase chain reaction assay suitable for large-scale screening of the prn type of the circulating strains. This method correctly identified the prn type of all tested 41 clinical isolates and two Finnish vaccine strains. The method is simple and reliable and provides an alternative for sequencing in pertussis research.


### Rapid Identification of *Bordetella pertussis* Pertactin Gene Variants Using LightCycler Real-Time Polymerase Chain Reaction Combined with Melting Curve Analysis and Gel Electrophoresis

**Johanna Mäkinen,*† Matti K. Viljanen,* Jussi Mertsola,†**
**Heikki Arvilommi,* and Qiushui He***
*National Public Health Institute, Department in Turku, Finland;
†Turku University Central Hospital, Turku, Finland

Recently, eight allelic variants of the pertactin gene (*prn1-8*) have been characterized in *Bordetella pertussis* strains isolated in Europe and the United States. It has been suggested that the divergence of the pertactin types of clinical isolates from those of the *B. pertussis* vaccine strains is a result of vaccine-driven evolution. Sequencing of the *prn,* which is relatively time-consuming, has so far been the only method for the differentiation of *prn* types. We have developed a rapid real-time polymerase chain reaction assay suitable for large-scale screening of the *prn* type of the circulating strains. This method correctly identified the *prn* type of all tested 41 clinical isolates and two Finnish vaccine strains. The method is simple and reliable and provides an alternative for sequencing in pertussis research.

*Bordetella pertussis* is the causative agent of pertussis (whooping cough), which is increasing in incidence in several countries despite high vaccination rates (1-5). One explanation for the increase might be the adaptation of *B. pertussis* bacteria to vaccine-induced immunity. Pertactin, a 69-kDa outer membrane protein, is an important virulence factor of *B. pertussis*. Because pertactin elicits protective immunity in animals and humans during vaccination (6-9), this protein is included in most new acellular pertussis vaccines. Pertactin contains two immunodominant regions, regions 1 and 2, comprising repeating units of five (GGxxP) or three (PQP) amino acids, respectively (10-12). It has been suggested that the number of the units is regulated through genetic recombination (12). Recently, eight allelic variants of the pertactin gene (*prn1-8*) have been characterized in *B. pertussis* strains isolated in Europe and the United States (12-16). Most of the allelic variation in *prn*1-5 are restricted to region 1, whereas *prn6-8* also show variation in region 2 (13). *prn1-3* are the predominant types, representing >90% of tested clinical isolates (12-16), whereas vaccine strains have exclusively *prn1* (12,14-16). Of 92 strains isolated between 1989 and 1999 in the United States, 30% harbored *prn1* and 70% *prn2* (14). In the Netherlands and Finland, approximately 10% of clinical strains isolated in the 1990s harbored *prn1* and 90%, *prn2* or *prn3* (12,15).

So far, the only means of determining the pertactin type has been polymerase chain reaction (PCR)-based sequencing of the *prn* gene, a relatively time-consuming and expensive method. To monitor the variation of clinical isolates on a large scale, a rapid and simple method is needed. The recent applications of fluorescence techniques to PCR allow real-time monitoring of accumulation of the amplified product and accurate analysis of the melting temperatures of either the amplified product itself or the attached hybridization probes (17-21). In the hybridization probe format the two independent, nonextendible, single-labeled oligonucleotide probes hybridize adjacently on the amplicon internal to the flanking PCR primers. After excitation by the light-emitting diode, a fluorescence resonance energy transfer (FRET) occurs from the donor dye to the acceptor dye, increasing the signal emitted by the acceptor dye (22).

We developed a simple method to characterize the pertactin variants (Figure 1). The strains with the frequent types, *prn1-5*, were first differentiated from strains with the rare types, *prn6-8*, by a real-time allele-specific amplification (ASA) assay. Strains representing *prn1-5* were further identified by a real-time PCR combined with the melting curve analysis of FRET probes and gel electrophoresis. Results were compared to those obtained by sequencing (15). The speed and simplicity of this approach make it an advantageous alternative to conventional sequencing of the *prn* gene.

### Materials and Methods

### Bacterial Strains and DNA Sequencing

Forty-one clinical *B. pertussis* isolates and 2 Finnish vaccine strains were selected from the strain collection of the Pertussis Reference Laboratory, National Public Health Institute, Turku, Finland. All 41 clinical isolates originated from Finland and were isolated from 1956 to 1996. The *prn*

Address for correspondence: Johanna Mäkinen, National Public Health Institute, Department in Turku, Kiinamyllynkatu 13, 20520 Turku, Finland; fax: 358-2-251-9254; e-mail: johanna.makinen@utu.fi

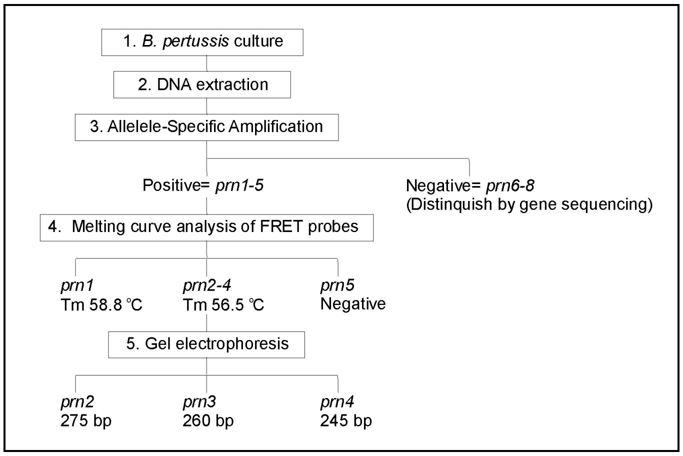

Figure 1. Workflow for typing *prn* alleles. The allele-specific amplification (ASA) assay (step number 3) and the fluorescence resonance energy transfer (FRET) probe assay (step number 4) each require approximately 1 hour.

```

                                               1595

QJF3       5´GCT GGT GCA GAC GCC AGT 3´

Prn 1-5      GCT GGT GCA GAC GCC ACT

Prn 6-8      --- --- --- --- --- --G

```

Figure 2. Partial sequence of the *prn* gene of *Bordetella pertussis*, showing the position of QJF3 primer. Consensus bases are shown with dashes, and the mismatched bases in the primer are underlined.

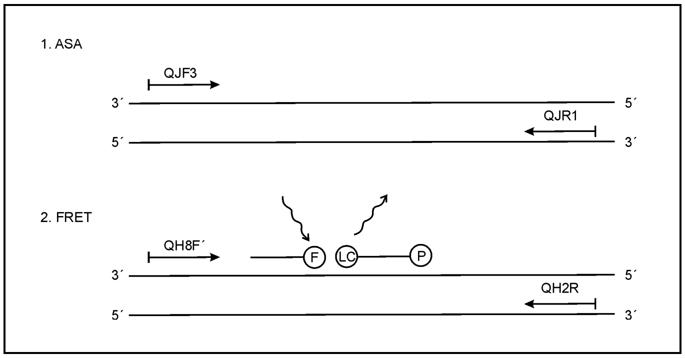

Figure 3. Schematic structure of the *prn1* gene, showing the position of the primers used in the allele-specific amplification assay (1.), and the primers and probes used in the fluorescence resonance energy transfer probe assay (2). Proportions of the gene are not drawn to scale. F = fluorescein; LC = LC red 640; and P = phosphate.

genes of these isolates and strains have been previously sequenced, and the *prn* sequences of 38 were published earlier (15). All the Finnish strains represented *prn1-4*. Strains B935 (AJ011016), 18323 (AJ132095), B567 (AJ133784), and B1092 (AJ133245) harbor *prn*5, *prn6*, *prn7*, and *prn8*, respectively.

Bacteria were cultivated on Regan-Lowe medium containing charcoal agar and defibrinated horse blood at 35°C for 3 days (23). Bacterial colonies on the plates were harvested for isolation of DNA. PCR-based sequencing was done as described previously (12).

### Primers and Probes

Primers for real-time ASA and the FRET probe assay were designed on the basis of the published sequence of the *prn* genes (10,12,15,16) and synthesized at Eurogentech, Seraing, Belgium (Table 1). Of primers used in ASA assay (Table 1) (Figures 2,3), QJF3 contained a specific mismatch G at the 3' end that did not complement the published sequences of any *prn* type. The T (boldfaced) at the 3'end of QJF3 (corresponding to the nucleotide 1595) was complementary to *prn1-5* but not to *prn6-8* to permit preferential amplification of the former types. The two mismatches at the 3'end of QJF3 would guarantee the absence of PCR amplification when the sequences of *prn6-8* are used as targets (24,25). The primers QJF3 and QJR1 define a 72-bp long PCR product. The primers QH8F' and QH2R used in the FRET probe assay define a 260-bp long PCR product. Based on earlier sequencing data, the calculated lengths of the PCR products were 260 bp, 275 bp, 260 bp, 245 bp, and 245 bp for *prn1*, *2*, *3*, *4*, and *5*, respectively. The FRET hybridization probes QJ1 and QJ2 were designed on the basis of the sequence of *prn1* to differentiate *prn1* from *prn3* (Table 1) (Figures 3,4). The boldfaced T of probe QJ2 is complementary to C to T transition specific for *prn*1 (corresponding to nucleotide 828) (13). Binding of probe QJ2 to *prn5* (compared to the *prn1-4*) will be hampered since no complementary sequence to the probe is available on *prn5* (Figure 4A, B). Probes were synthesized at TIB Molbiol, Berlin, Germany. QJ1 (used as the donor probe in FRET technology) was labeled with fluorescein at the 3' end. QJ2 was labeled with LightCycler Red 640 at the 5'end and phosphorylated at the 3' end; this was used as the acceptor probe in the FRET (Table 1) (Figure 4A).

Table 1. Primers and probes used in study of *Bordetella pertussis* pertactin gene variants

| Primer/ probe | Sequence (5'-3')[a] | Position[b] |
|---|---|---|
| QJF3[c] | GCT GGT GCA GAC GCC AG**T** | 1578-1595 |
| QJR1[c] | CCG ATA TCG ACC TTG CC | 1649-1633 |
| QH8F[d] | CTG CAG CGC GCG ACG ATA | 757-774 |
| QH2R[d] | ATT GCC GTG CGG TGC GGA CAA | 1026-1006 |
| QJ1[e] | CCG GCG GTG CGG TTC C-**F** | 809-824 |
| QJ2[e] | **LC Red 640**-CGG **T**GG TGC GGT TCC C-**P** | 825-840 |

[a]Modifications of primer or probe are boldfaced or underlined.
[b]Position numbers indicate the position of bases relative to the first start codon of *prn1*.
[c]Primers used in the real-time allele-specific amplification. QJF3 contained a specific mismatch G (underlined) at the 3' end that does not complement the published sequences of any *prn* type. The T (boldfaced) at the 3' end (corresponding to the nucleotide 1595) is complementary to *prn1-5*. Primer QJF3 has two mismatches with *prn6-8*.
[d]Primers used in fluorescence resonance energy transfer (FRET) probe assay.
[e]Probes used in FRET probe assay. Boldfaced T is complementary to C to T transition specific for *prn1.* QJ1 was labeled with fluorescein at the 3' end and QJ2 with LC Red at 5' end and phosphorylated at the 3' end.

### DNA Preparation

DNA was extracted from bacterial colonies by using the DNA Isolation Kit for Blood/Bone Marrow/Tissue (Roche Diagnostics, Mannheim, Germany) according to the

Figure 4A. Nucleotide sequences of polymorphic regions of different types of the *prn* gene and the sequences of the fluorescence resonance energy transfer probes aligned to their hybridization positions in the *prn* gene. Number 790 refers to the position of bases relative to the first start codon of *prn1*. Underlined regions represent repeats in the sequence. B. Amino acid sequences of polymorphic regions of different pertactin types. Dashes indicate caps in the sequence.

manufacturer's instructions. Extracted DNA concentrations were measured with a GeneQuant spectrophotometer (Pharmacia Biotech, NJ, USA). DNA concentrations in all samples were adjusted to 3 ng/µL. DNA preparations were stored at -20°C.

### Allele-Specific Amplification (ASA)

ASA PCR, which distinguishes between *prn1-5* and *prn6-8,* was performed in a fluorescence temperature cycler (LightCycler, Roche). The PCR reaction mixture was optimized for the LightCycler and amplified according to the manufacturer's protocol. The final volume of 20 µL contained 2 µL of LightCycler-DNA Master SYBR Green I (containing Taq DNA polymerase, reaction buffer, deoxynucleoside triphosphate (dNTP) mix and dsDNA binding dye SYBR Green I), 4 mM $MgCl_2$ (Roche), 8 pmol of the primers QJF3 and QJR1, 5% dimethyl sulfoxide (Merck, Darmstadt, Germany), and 2 µL of 3-ng/µL sample DNA. A negative control without DNA and a positive control that contained 6 ng of the DNA from strain 1772 (*prn1*) were included in each run. The amplification protocol consisted of the initial denaturation step at 94°C for 30 seconds, 30 cycles of denaturation at 95°C for 1 second, annealing at 62°C for 5 seconds, and extension at 72°C for 4 seconds. The temperature transition rate was 20°C per second. Fluorescence was measured at the end of each extension step at 530 nm. The increase in the fluorescence signal correlates to the accumulation of PCR product (19,22).

After amplification, melting curve analysis of the PCR product was used to differentiate between specific and nonspecific amplification products. Melting curve was acquired by heating the product at 20°C/seconds to 95°C, cooling it at 20°C/seconds to 55°C for 30 seconds, and slowly heating it at 0.1°C/seconds to 94°C under continuous fluorescence monitoring. Melting curve analysis was accomplished with LightCycler software. As the temperature reaches the specific Tm of the PCR product, the double-stranded product is rendered into the single-stranded form. A rapid loss of fluorescence can be observed as the double-stranded DNA binding dye SYBR green I detaches from the PCR products. The change in fluorescence signal intensity is then plotted as the negative derivative of fluorescence versus temperature (–dF/dT vs T graphs) to obtain the characteristic melting peaks. In constant reaction conditions (salt concentration and the like), the position of the melting curve peak (Tm) is a function of the GC/AT ratio, length, and nucleotide sequence of the PCR product (26). Melting curve analysis has been successfully used in the differentiation of PCR products with a difference of even <2°C in the Tm (18,26).

### Hybridization Probe Assay

The hybridization probe assay was carried out by using the FRET probe format of LightCycler. The PCR reaction mixture was optimized for the LightCycler and amplified according to the manufacturer's protocol. The final volume of 20 µL contained 2 µL of LightCycler-DNA Master Hybridization Probes (containing Taq DNA polymerase, reaction buffer, and dNTP mix), 3 mM $MgCl_2$ (Roche), 1.5 pmol of the FRET probes QJ1 and QJ2, 8 pmol of the primers QH8F' and QH2R, 220 ng of TaqStart antibody (ClonTech, CA, USA), 10% dimethyl sulfoxide (Merck), and 2 µL of sample DNA. A negative control without DNA and two positive controls representing *prn1* and *3* were included in each run. The temperature profile of the real-time PCR included an initial denaturation step at 94°C for 120 seconds followed by 40 cycles of denaturation at 94°C for 2 seconds, annealing at 55°C for 10 seconds, and extension at 72°C for 12 seconds. The temperature transition rate was 20°C/s. Fluorescence was measured at 640 nm at the end of the annealing step of each cycle to monitor the accumulation of PCR product.

After amplification, a melting curve was acquired by heating the product at 20°C/seconds to 95°C, cooling it at 20°C/seconds to 42°C for 120 seconds, and slowly heating it at 0.1°C/seconds to 80°C under continuous fluorescence monitoring. Melting curve analysis was accomplished using LightCycler software. In the hybridization probe format, the rapid loss of fluorescence is observed when the temperature reaches the Tm of the probes. The two adjacently bound probes dissociate from their complementary target, which prevents the fluorescence resonance energy transfer. Melting curve analysis allowed us to discriminate the specific binding of the hybridization probes to the amplified segment of the *prn1* from their less specific binding to the amplified segments of the other *prn* types.

### Analysis of the FRET Hybridization Probe Assay PCR Products by Gel Electrophoresis

A 20-µL volume of LightCycler PCR product from the hybridization probe assay was removed from the capillary by removing the cap, placing the capillary upside down in an empty Eppendorf tube, and centrifuging for 5 seconds. A 10-µL volume of the PCR product was run (100 V for 3 hours) in a 3% molecular screening (MS) agarose gel (Roche Diagnostics) together with a 100-bp DNA ladder (Amersham). According to the manufacturer, the resolution characteristics of MS agarose enable separation of fragments that differ in size by as little as 4 bp. After being stained with ethidium bromide, the bands in the gel were visualized and photographed under UV light. To avoid PCR contamination, three separate rooms were used for preparing the PCR mixtures, performing PCR reactions, and analyzing PCR products.

### Statistical Analysis

The Student *t* test was used to analyze statistical significance. All p values corresponded to two-tailed tests, and $p < 0.05$ was considered significant.

### Results

### Differentiation of *prn1-5* from *6-8*

The ASA assay was used as a screening method to differentiate the frequent *prn* types (*prn1-5*) from rare types (*prn6-8*). When compared to previous sequencing data (12), all type strains and clinical isolates were correctly categorized by this assay. The mean Tm of the PCR products derived from the *prn1-5* strains was 83.4°C (standard deviation [SD] 0,54) (Figure 5). There was no specific amplification from the strains with *prn6-8*. The nonspecific products, such as primer dimers, melt below 80°C and were differentiated from the specific products by the melting curve analysis. These results were also confirmed by gel electrophoresis.

### Melting Curve Analysis of Hybridization Probe Assay

Strains that were found to harbor *prn1-5* in the screening were further analyzed by the combination of hybridization probe assay and gel electrophoresis. FRET probes were designed as complementary to the *prn1* gene. The Tms and results of the melting curve analyses of the strains with the *prn1* gene differed markedly from those of strains with *prn2, prn3*, and *prn4* (Figure 6). All nine strains harboring the *prn1* gene showed an abrupt decrease in the fluorescence signal (Table 2) and a melting peak (Figure 6) at 58.78°C (SD 0.26). The corresponding melting peak was observed at the same temperature in all nine *prn1* strains. Although probes did not remain bound to PCR products of *prn2, prn3*, and *prn4* when the fluorescence signal was measured at the end of the annealing cycle at 55°C, probes bound to those products at the beginning of the melting analysis at 42°C, and Tm and the area under the melting curve (AUC) could also

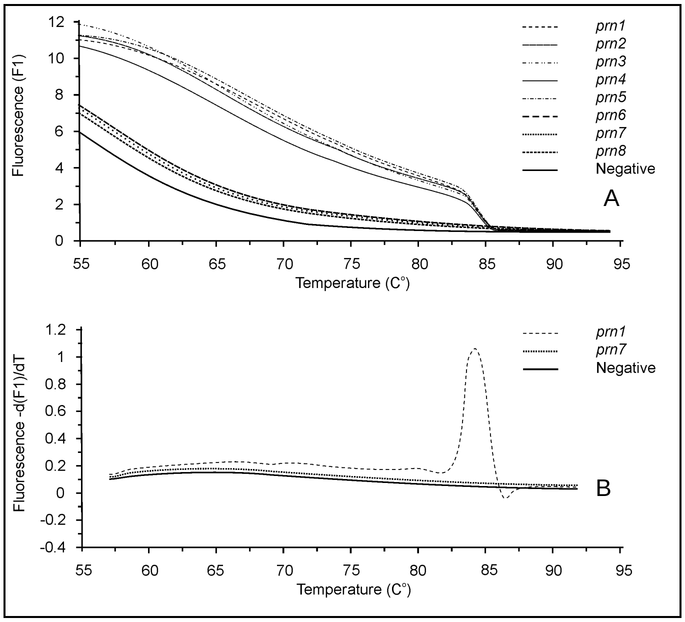

Figure 5A. Melting curves from the allele-specific amplification assay, showing the presence of amplified products from *prn1-5* and the absence of amplification from *prn6 -8* and the negative control. B. Corresponding melting peaks derived from the melting curve. *Prn1* represents the *prn1-5* types; *prn7* represents *prn6-8* types and the negative control.

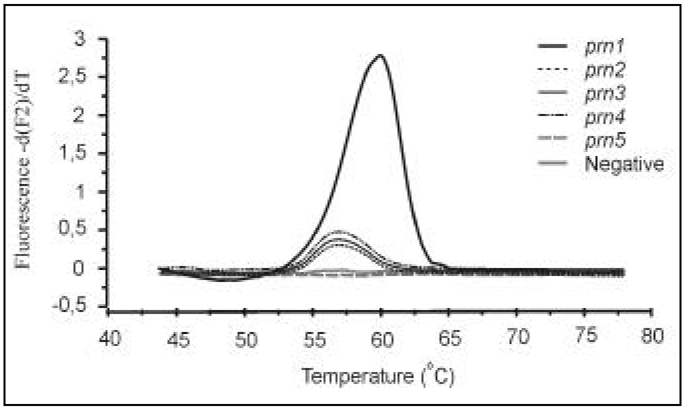

Figure 6. Curves showing the dissociation of fluorescence resonance energy transfer probe assay probes from the polymerase chain reaction products of different *prn* types. Negative control includes all reagents but no template DNA.

be determined for these products. As expected, the Tm of FRET probes bound to the PCR products derived from *prn2, prn3*, and *prn4* was 2°C lower (56.49°C) than that of the FRET probes bound to the PCR product of *prn1* (all p values for differences between the Tm of *prn1* and that of the other *prn* types were <0.0001) (Table 2) (Figure 6). The AUC of the hybridization probe melting curve of *prn1* was approximately 20 times larger than that of the melting curve of the *prn2, prn3*, and *prn4* (all p values <0.0001) (Table 2) (Figure 6). There was no detectable binding of FRET probes to the PCR products derived from the *prn5* strain (Figure 6). DNA isolated from the strain with *prn5* (together with DNAs from strains representing *prn1-4* that served as controls) was tested in triplicate and with different DNA concentrations with the same result. In contrast to *prn2-4*, there was no measurable melting temperature and no AUC from the DNA isolated from the strain with *prn5*, although the PCR product was seen (245-bp long on the electrophoresis gel) (Figure

Table 2. Comparison of the results from sequencing and the hybridization probe assay in the determination of the pertactin gene type of *Bordetella pertussis* strains

| Pertactin allele type[a] | No. of isolates | Melting temperature | | Melting curve | |
|---|---|---|---|---|---|
| | | Mean | SD | Area | SD |
| 1 | 9 | 58.78[b] | 0.26 | 21.64[b] | 5.64 |
| 2 | 25 | 56.31 | 0.44 | 1.25 | 0.30 |
| 3 | 4 | 56.59 | 0.09 | 1.35 | 0.26 |
| 4 | 5 | 56.57 | 0.29 | 1.43 | 0.25 |
| 5 | 1 | - | - | - | - |

[a]Allele type determined by sequencing. Of the strains representing *prn1-4*, 41 were Finnish clinical isolates, and 2 were Finnish vaccine strains.
[b]All P values were <0.0001 when *prn1* was compared to *prn2*, *prn*3, or *prn4*.

7). In this setting, therefore, a sample that remained totally negative (no measurable melting temperature and no AUC) in the hybridization probe assay but was characterized as *prn1-5* type strain by the ASA assay was considered to harbor *prn5*.

### Gel Electrophoresis of PCR Products

Calculated sizes of PCR products were 260 bp, 275 bp, 260 bp, 245 bp, and 245 bp for *prn1* to *5*, respectively. PCR products of the different *prn* types behaved in gel electrophoresis as expected on the basis of their calculated sizes (Figure 7). Thus, *prn2*, *prn3*, and *prn4* could be easily differentiated by gel electrophoresis when the *prn1* and *prn5* were identified by the melting curve analysis of hybridization probes.

### Identification of the *prn* Type of *B. pertussis* Isolates and Vaccine Strains

The *prn* types of all tested 41 Finnish clinical *B. pertussis* isolates and 2 Finnish vaccine strains were identified correctly when compared to types defined by sequencing (Table 2). None of these strains was found to harbor *prn5-8*.

### Discussion

Real-time PCR combined with melting curve analysis of FRET probes and gel electrophoresis of PCR products proved to be an alternative to sequencing in the determination of the pertactin gene types of *B. pertussis*. The method was reliable and accurate, as evidenced by the correct identification of the *prn* type of all tested 41 clinical *B. pertussis* isolates, two vaccine strains, and the four reference strains. The advantage of this approach over sequencing is that the whole procedure from nucleic acid extraction to gel electrophoresis can be completed within 1 day. The disadvantages of the method are that the novel genotypes can be missed and the method does not differentiate *prn6-8* from each other.

The low intra- and inter-assay variation coefficients of melting temperatures show that the technical principles of LightCycler allow consistent temperature and fluorescence measurement conditions for the reaction capillaries. This is a definite advantage over the corresponding instruments using the microwell plate format, which requires intrinsic correction to compensate for technical variation between reaction wells.

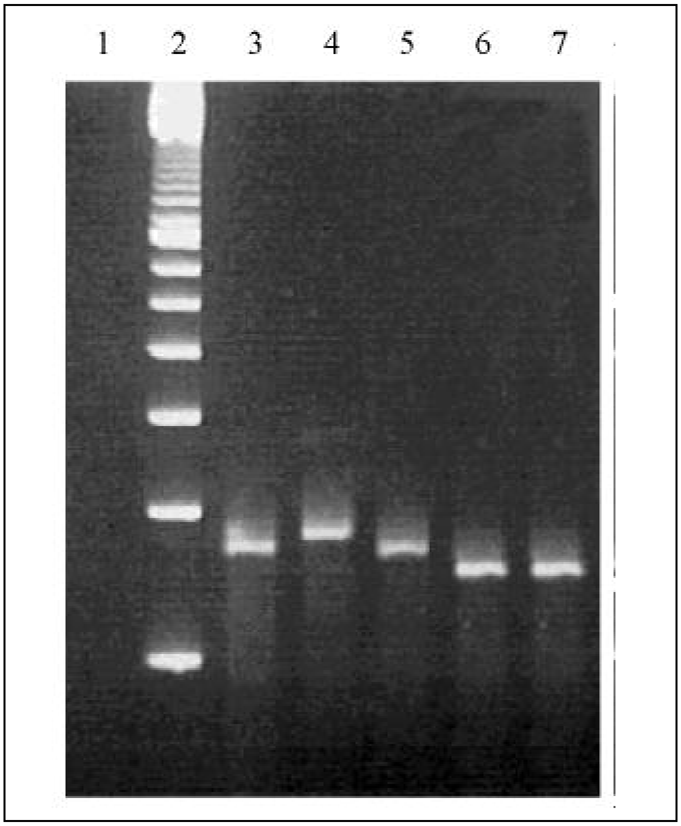

Figure 7. Ethidium bromide stained 3% molecular screening agarose gel containing *Bordetella pertussis* DNA amplified with primers QH8F' and QH2R. Lanes: 1, negative control including all reagents but no template DNA; 2, 100-bp ladder; 3, *B. pertussis* strain 1772 of type *prn1* (260 bp); 4, *Bordetella pertussis* clinical isolate of type *prn2* (275 bp); 5, *B. pertussis* clinical isolate of type *prn3* (260 bp); 6, *B. pertussis* clinical isolate of type *prn4* (245 bp); and 7, *B. pertussis* type *prn5* (245 bp).

In this study, a real-time ASA assay was used as a screening method to first differentiate the frequent *prn* types *prn1-5* from the rare types *prn6-8* (13). In ASA assay, when primers designed to be specific for either wild-type or the mutant allele are used, results depend on the presence or absence of amplification. In this study the PCR amplification by the primers specifically designed for the allele *prn1-5* took place with DNAs extracted from *prn1-5* strains but not with those isolated from strains representing *prn6-8*. When ASA reactions occur in a fluorescence thermal cycler such as LightCycler, accumulation of the PCR product can be monitored in real-time. The analysis of specific melting temperatures further confirms the identity of the amplified products.

The FRET probes were specifically designed to identify *prn1* so that the strains representing the vaccine type *prn* could be rapidly detected. The FRET probes also enabled differentiation of *prn1* from *prn3*, the two *prn* types that cannot be differentiated on the basis of the size of the PCR product. The *prn1* sequence contains an additional C to T transition (corresponding to nucleotide 828) that makes it possible to design probes that are specific for just one *prn* type. The probes did not bind to the PCR products of *prn2*, *prn3*, *prn4*, and *prn5* in the fluorescence measurement phase of the PCR cycle at 55°C. Therefore, no signal was obtained in the real-time PCR from DNA of bacteria having these *prn* types. However, probes did bind to the PCR products of *prn2*, *3*, and *4* at the beginning of the melting analysis at 42°C, and the Tm and AUC values could also be determined for these products. Their Tm was >2°C lower and AUC approximately 20 times smaller than those of the PCR product of *prn1*. These differences clearly reflect the lower sequence compatibility of the probes for *prn2*, *prn3*, and *prn4* than for *prn1*. As expected, the Tms of *prn2*, *prn3*, and *prn4* were almost identical because the target sequences of the probes in these *prn* types were the same. *Prn5* has the same number of repeats as *prn4* does, which makes their PCR products the same length. However, the difference between these two is that *prn4* has two repeats of "GGAVP," as do *prn2* and *prn3*, whereas *prn5* has only one such repeat. *Prn5* is the only type to which there was no detectable binding of the FRET probes during the amplification phase or the melting curve analysis. This property could be used to differentiate *prn5* from *prn4*.

The difference in size of the PCR products derived from the *prn2*, *prn3*, and *prn4* (or *prn*5) is 15 bp. To assure the correct identification of the pertactin types, a gel with a high-resolution power is needed for the electrophoresis. In this study molecular screening agarose gel was used, since the resolution characteristics of this agarose enable separation of fragments that differ in size by as little as 4 bp.

Recent data suggest that *B. pertussis* strains having different *prn* types are circulating in Europe and the United States. The predominant types representing >90% of the tested clinical isolates are *prn1-3* (12-16).

In the United States, all strains isolated before 1974 harbored *prn1* (14), the *prn* type of the strains included in conventional whole-cell vaccines and in the new acellular vaccines. However, nonvaccine *prn* types gradually replaced the vaccine types in later years, and approximately 30% of strains isolated between 1989 and 1999 were *prn1*. Similar trends of frequency in strain types were also seen in European countries. The method described here is suitable for monitoring the frequency of the strain types of clinical isolates. The strains representing *prn1* can be detected by running the two PCR reactions (ASA and hybridization probe assay), and the results can be obtained within 2 hours. When the strains that do not represent vaccine strain type need to be clarified, gel electrophoresis of PCR products can be performed. To combat pertussis and to design more effective vaccines, the variation of pertactin and other virulence factors of the *B. pertussis* strains circulating in the population has to be monitored. It is possible that the antigenic variation is a result of vaccine-driven evolution, possibly protecting the bacteria from the attacks of the host's specific immune response. The method described here is convenient for large-scale screening of pertactin variation in *B. pertussis* isolates. Data obtained by large-scale screening provide the epidemiologic picture of the circulating strains. This information may further help in vaccine formulation, which enables more efficient protection against pertussis. It is also possible to use a similar approach to detect the variation in pertussis toxin gene that has been already characterized, or in studies on genetic variation in any species.

### Acknowledgments

We thank Tuula Rantasalo and Birgitta Aittanen for technical assistance; Erkki Nieminen for help in preparing figures; Simo Merne for revising the manuscript; Olfert Landt for designing the probes; and Nicole Guiso, Hans Hallander, and Frits Mooi for providing strains.

The Academy of Finland and the Special Governmental Fund for University Hospitals financially supported this work.

Mrs. Mäkinen née Pietilä is a researcher at the Finnish Pertussis Reference Laboratory, National Public Health Institute of Finland. Her research interests focus on the characterization and detection of antigenic variation in *Bordetella* organisms.

### References

1. Andrews R, Herceq A, Roberts C. Pertussis notifications in Australia. Commun Dis Intell 1997;21:145-8.
2. Bass JW, Wittler RR. Return of epidemic pertussis in the United States. Pediatr Infect Dis J 1994;13:343-5.
3. Bass JW, Stephenson SR. The return of pertussis. Pediatr Infect Dis J 1987;6:141-4.
4. de Melker HE, Conyn-van Spaendock MAE, Rümke HC, van Wijngaarden JK, Mooi FR, Schellekens JFP. Pertussis in the Netherlands: an outbreak despite high levels of immunization with whole cell vaccine. Emerg Infect Dis 1997;3:175-8.
5. DeSerres G, Boulianne N, Douville Fradet M, Duval B. Pertussis in Quebec: ongoing epidemic since the late 1980s. Can Commun Dis Rep 1995;15:45-8.
6. Brennan MJ, Li ZM, Cowell JL, Bisher ME, Steven AC, Novotny P, et al. Identification of a 69-kilodalton nonfimbrial protein as an agglutinogen of *Bordetella pertussis*. Infect Immun 1988;56:3189-95.
7. Cherry JD, Gornbein J, Heininger U, Stehr K. A search for serologic correlates of immunity to *Bordetella pertussis* cough illness. Vaccine 1998;199:1901-6.
8. Shahin RD, Brennan MJ, Li ZM, Meade BD, Manclark CR. Characterization of the protective capacity and immunogenicity of the 69-kD outer membrane protein of *Bordetella pertussis*. J Exp Med 1990;171:63-73.
9. Storsaeter J, Hallander HO, Gustafsson L, Olin P. Levels of anti-pertussis antibodies related to protection after household exposure to *Bordetella pertussis*. Vaccine 1998;16:1907-16.
10. Charles IG, Dougan G, Pickard D, Chatfield S, Smith M, Novotny P, et al. Molecular cloning and characterization of protective outer membrane protein P.69 from *Bordetella pertussis*. Proc Natl Acad Sci U S A 1989;86:3554-8.
11. Charles IG, Li J, Roberts M, Beesley K, Romanos M, Pickard DJ, et al. Identification and characterization of a protective immunodominant B cell epitope of pertactin (P.69) from *Bordetella pertussis*. Eur J Immunol 1991;21:1147-53.
12. Mooi FR, van Oirschot H, Heuvelman K, van der Heide HGJ, Gaastra W, Willems RJL. Polymorphism in the *Bordetella pertussis* virulence factors P.69/pertactin and pertussis toxin in the Netherlands: temporal trends and evidence for vaccine-driven evolution. Infect Immun 1998;66:670-5.
13. Mooi FR, Hallander H, Wirsing von König CH, Hoet B, Guiso N. Epidemiological typing of *Bordetella pertussis* isolates: recommendations for a standard methodology. Eur J Clin Microbiol Infect Dis 2000;19:174-81.
14. Cassiday P, Sanden G, Heuvelman K, Mooi F, Bisgard KM, Popovic T. Polymorphism in *Bordetella pertussis* pertactin and pertussis toxin virulence factors in the United States, 1935-1999. J Infect Dis 2000;182:1402-8.
15. Mooi FR, He Q, van Oirschot H, Mertsola J. Variation in the *Bordetella pertussis* virulence factors pertussis toxin and pertactin in vaccine strains and clinical isolates in Finland. Infect Immun 1999;67:3133-4.
16. Mastrantonio P, Spigaglia P, van Oirschot H, van der Heide HGJ, Heuvelman K, Stefanelli P, et al. Antigenic variants in *Bordetella pertussis* strains isolated from vaccinated and unvaccinated children. Microbiology 1999;145:2069-75.
17. Von Ahsen N, Oellerich M, Armstrong VW, Schütz E. Application of a thermodynamic nearest-neighbour model to estimate nucleic acid stability and optimize probe design: prediction of melting points of multiple mutations of apolipoprotein B-3500 and factor V with a hybridization probe genotyping assay on the Light Cycler. Clin Chem 1999;45:2094-101.

18. Bohling SD, Wittwer CT, King TC, Elenitoba-Johnson KSJ. Fluorescence melting curve analysis for the detection of the bcl-1/JH translocation in mantle cell lymphoma. Lab Invest 1999;79:337-45.
19. Nitsche A, Steuer N, Schmidt CA, Landt O, Siegert W. Different real-time PCR formats compared for the quantitative detection of human cytomegalovirus DNA. Clin Chem 1999;45:1932-7.
20. Wittwer CT, Ririe KM, Andrew RV, David DA, Gundry RA, Balis UJ. The LightCycler: a microvolume multisample fluorimeter with rapid temperature control. Biotechniques 1997;22:176-81.
21. Pietilä J, He Q, Oksi J, Viljanen MK. Rapid differentiation of *Borrelia garinii* from *Borrelia afzelii* and *Borrelia burgdorferii* sensu stricto by LightCycler fluorescence melting curve analysis of a PCR product of the *recA* gene. J Clin Microbiol 2000;38:2756-9.
22. Wittwer CT, Herrman MG, Moss AA, Rasmussen RP. Continuous fluorescence monitoring of rapid cycle DNA amplification. Biotechniques 1997;22:130-8.
23. He Q, Mertsola J, Soini H, Skurnik M, Ruuskanen O, Viljanen MK. Comparison of polymerase chain reaction with culture and enzyme immunoassay for diagnosis of pertussis. J Clin Microbiol 1993;31:642-5.
24. Espinosa de los Monteros LE, Galán JC, Gutiérrez M, Samper S, Marín JFG, Martín C, et al. Allele-specific PCR method based on *pncA* and *oxyR* sequences for distinguishing *Mycobacterium bovis* from *M. tuberculosis*: intraspecific *M. bovis pncA* sequence polymorphism. J Clin Microbiol 1998;36:239-42.
25. Cebula TA, Payne WL, Feng P. Simultaneous identification of strains of *Escherichia coli* serotype O157:H7 and their Shiga-like toxin type by mismatch amplification mutation assay-multiplex PCR. J Clin Microbiol 1995;33:248-50.
26. Ririe KM, Rasmussen RP, Wittwer CT. Product differentiation by analysis of DNA melting curves during the polymerase chain reaction. Anal Biochem 1997;245:154-60.
